# Comparative plastome data analysis of Dendrosicyos socotranus and Corallocarpus boehmii

**DOI:** 10.6026/97320630017662

**Published:** 2021-07-31

**Authors:** Mohammad Ajmal Ali, Khalid Mashay Al-Anazi, Fahad Al-Hemaid, Waquar Akhtar Ansari, Joongku Lee

**Affiliations:** 1 Department of Botany and Microbiology, College of Science, King Saud University, Riyadh-11451, Saudi Arabia; 2 Genetics Laboratory,Department of Zoology, College of Science, King Saud University, Riyadh-11451, Saudi Arabia; 3 Department of Botany, Banaras Hindu University, Varanasi - 221005, India; 4 Department of Environment and Forest Resources, Chungnam National University, Daejeon 34134, Republic of Korea

**Keywords:** Dendrosicyos socotrana, Corallocarpus boehmii, Coniandreae, Cucurbitaceae, Plastome

## Abstract

Comparison of the Dendrosicyos socotranus and Corallocarpus boehmii (tribe Coniandreae, family Cucurbitaceae) plastome data was of interest. Data on RNA, tRNA, GC%, plastome size, CDS and pseudogene were tabulated for the two species. The total length of 1,57,380 bp and 1,58,744 bp which includes LSC, SSC, IRa, and IRb, while their GC content was 37.1% and 37% respectively. The variation in the length of genes e.g. ndhD, ndhI, rpl22, rpoC2, rps16, rps19, rps8, ycf1and ycf2 noted. Data help to document the genetic differences between usual (climber) with those of tree cucurbits.

## Background:

The Cucurbitaceae, also called cucurbits or the gourd family, are consisting of 1000 species under 98 genera. The family is distributed throughout the world mostly in tropical and subtropical region [1]. It is considered as one of the most diversified plant family with economical [1] and pharmacological significance [2]. The plants of this family are herbaceous annual climbers, vines or woody perennial lianas, mostly tendril-bearing monoecious or dioecious [1] except the bottle-trunked succulent tree e.g. Dendrosicyos socotrana which is characterized through its characteristic of distended water capturing trunk, pendulous branches, tendrils absent, monoecious long yellow flowers which forms tubular hypanthium, flowers individual or in small fascicles, three stamens, and fruits are ellipsoid in shape, smooth, green and turn brick-red when ripen [1]. The massive advancement in next generation sequencing and analyses during the last decade has helped plastome sequencing easier and affordable. The plastome data are useful in understanding the tree of life and biotechnological application [3- 4]. Therefore, the comparative plastome data analysis of two members of the tribe Coniandreae (Cucurbitaceae) i.e. Dendrosicyos socotranus and Corallocarpus boehmii was of interest to document the genetic differences in the plastome between usual (climber) cucurbits with those of tree cucurbits.

## Materials & Methods:

Dendrosicyos socotranus and Corallocarpus boehmii fasta plastome sequence data were downloaded from NCBI (Table 1 - see PDF). The plastome annotation was performed using default option of GeSeq [5] as shown in Figure 2. The annotated data were further tabulated and plastome size, GC%, CDS, rRNA, tRNA, and pseudogene were compared.

## Results and Discussion:

**Figure 1:**
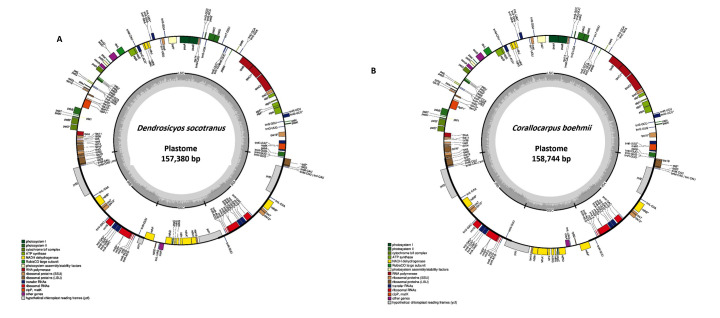
The genes belonging to different functional groups depicted in plastome map of D. socotranus and C. boehmii are shown in different colors.

**Figure 2:**
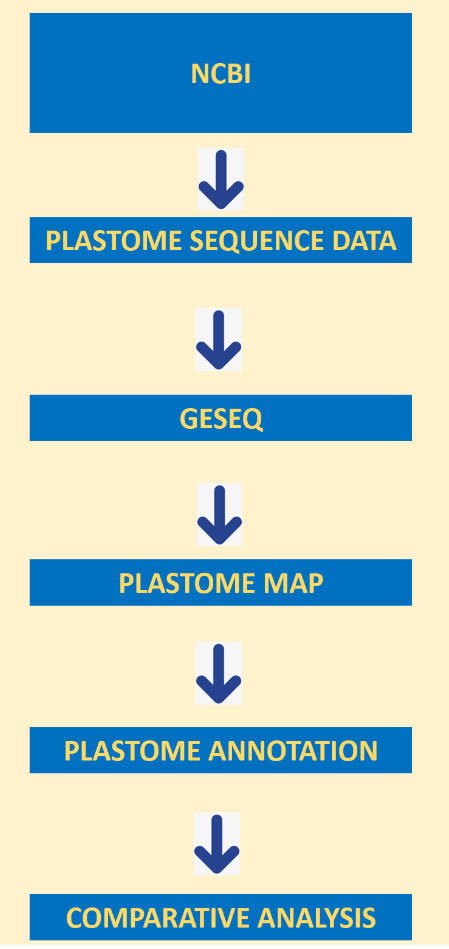
The methodology flowchart.

D. socotranus and C. boehmi annotated plastome maps as a conserved circular structure. It includes LSC, SSC, IRa, and IRb with total length 1,57,380 bp and 1,58,744 bp (Figure 1 A-B) with GC content 37.1% and 37%, respectively similar to other angiosperms [4]. In D. socotranus, the plastome carried 130 genes, which includes 84 CDS, 37 tRNA, and 8 rRNA genes (Figure 1A). The comparative analysis of D. socotranus with C. boehmi plastome exhibited the variation of gene length e.g. ndhD (NADH dehydrogenase subunit D), ndhI (NADH dehydrogenase subunit I), rpl22 (ribosomal protein L22), rpoC2 (RNA polymerase beta subunit), rps16 (ribosomal protein S16), rps19 (ribosomal protein S19), rps8 (ribosomal protein S8), ycf1 (Ycf1 protein), ycf2 (Ycf2 protein) (Table 2 - see PDF). Additionally, the C. boehmi carry ycf1 (1890 bp and 402 bp), while in D. socotranus it was 402 bp. ycf1 and ycf2 genes are coded by two longest ORF (open reading frame) in plastome, ycf1 gene is noted to be involved in the ATP-dependent vacuolar transport of bilirubin and glutathione conjugates [6-7], in case of angiosperms it has been considered as a most potential plastid DNA barcoding gene [8].

The plastome is considered as one of the distinctive organelle of plant cells as it carries own unique genome, carrying genes largely linked to housekeeping activities and photosynthesis. Its highly conserved sequence and high copy number in cells make the plastome an easily available resource of significant phylogenetic messages. Advancement in the next-generation sequencing (NGS) as well as bioinformatics tools used for the NGS data analysis in recent years [3-9], made easy to study in detail about the plastome [4] and whole genome [10] sequencing. This has transformed the comprehension of development of plant genomes [11]. The present study is elucidating the plastome characteristics of bottle-trunked succulent cucurbit tree. The exploration of NCBI Organelle Genome Resources exposed a total number of plastome sequence data of only 64 species (out of 1,000 species) of the family Cucurbitaceae. The plastome study of large number of cucurbits is yet to be explored. Nevertheless, the easiness of NGS and analyses would bring more plastome data, nuclear genome and transcriptome data, might resolve the genetic divergence between climbing cucurbits with those of tree cucurbits.

## Conclusion:

We document the variation in the length of genes e.g. ndhD, ndhI, rpl22, rpoC2, rps16, rps19, rps8, ycf1 and ycf2. Data help to document the genetic differences between usual (climber) with those of tree cucurbits.
